# Supplementary material for: Structural and mechanistic insights into gating and allosteric modulation of GluN1–GluN3A NMDA receptors
Source: Nat Struct Mol Biol. 2026 Aug 28;33(9):1299–310. doi: 10.1038/s41594-026-01866-9 (PMC13561882; doi:10.1038/s41594-026-01866-9)
Supplement: Supplementary file 1 [file 41594_2026_1866_MOESM1_ESM.pdf]

nature structural & molecular biology

Supplementary information https://doi.org/10.1038/s41594-026-01866-9

Structural and mechanistic insights into gating and allosteric modulation of GluN1–GluN3A NMDA receptors

In the format provided by the authors and unedited

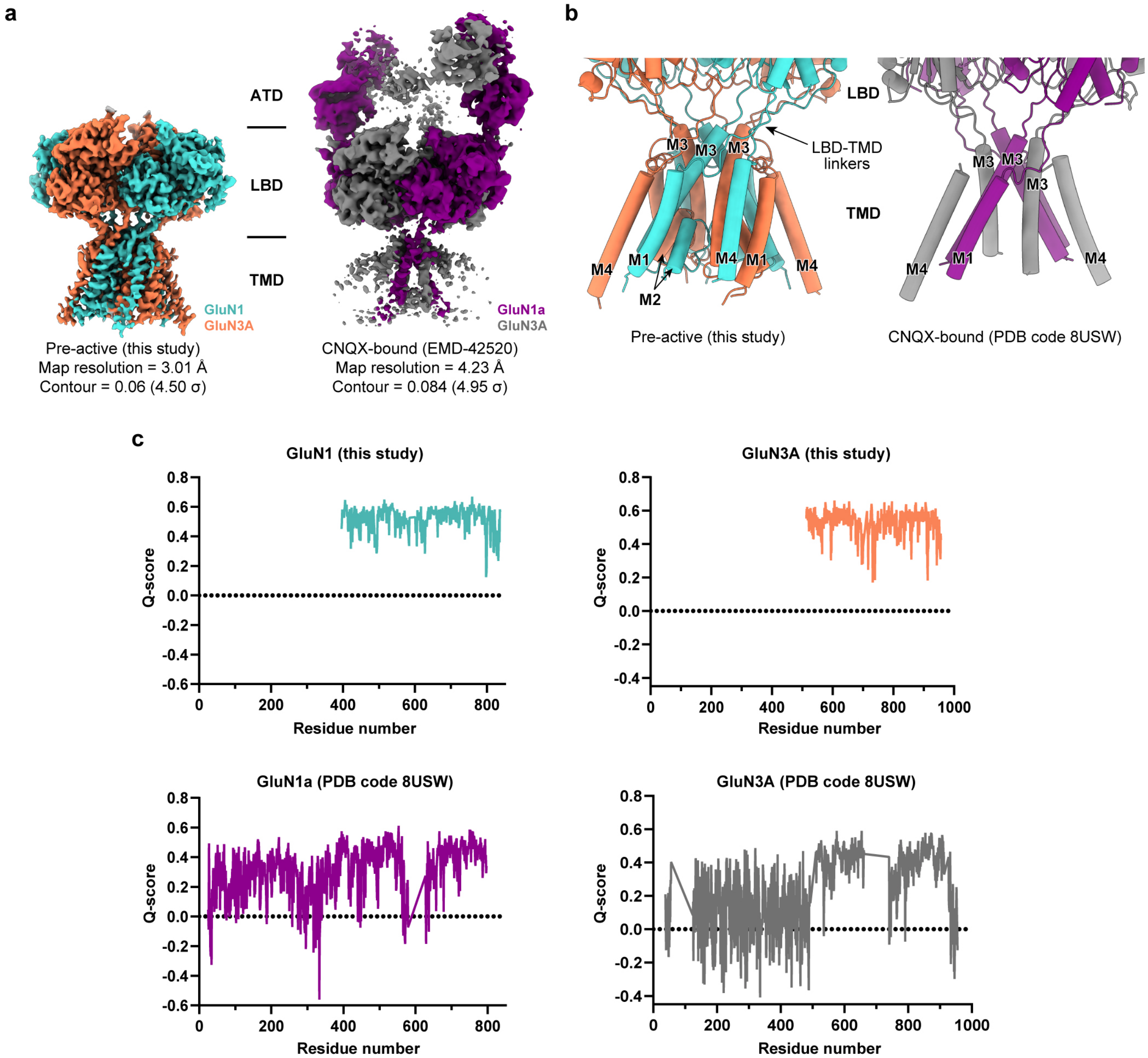

Supplementary Fig. 1: Comparison of GluN1/GluN3A cryo-EM maps and models in this study and in a previous report. a, Cryo-EM densities of the LBD-TMD layer in the pre-active state revealed in this study (left) and the CNQX-bound engineered GluN1a/GluN3A reported by Michalski K. and Furukawa H., Sci. Adv., 2024 (right). The map resolutions and volume levels for the views are indicated. **b**, Structural models including the lower lobe of the LBD and TMD layers show the nearly completely resolved TM helices and LBD-TMD linkers in the pre-active structure, whereas the corresponding elements are incompletely modeled or missing in the CNQX-bound structure. **c**, Q-score plots showing the resolvability of individual atoms in each density map, with a score of +1 indicating perfect similarity to a Gaussian function and a score closer to 0 indicating low similarity. A negative Q-score indicates that the atomic model is not supported by the cryo-EM density at that position, suggesting missing or incorrectly modeled structural features in the map. See more detailed descriptions in the main text.

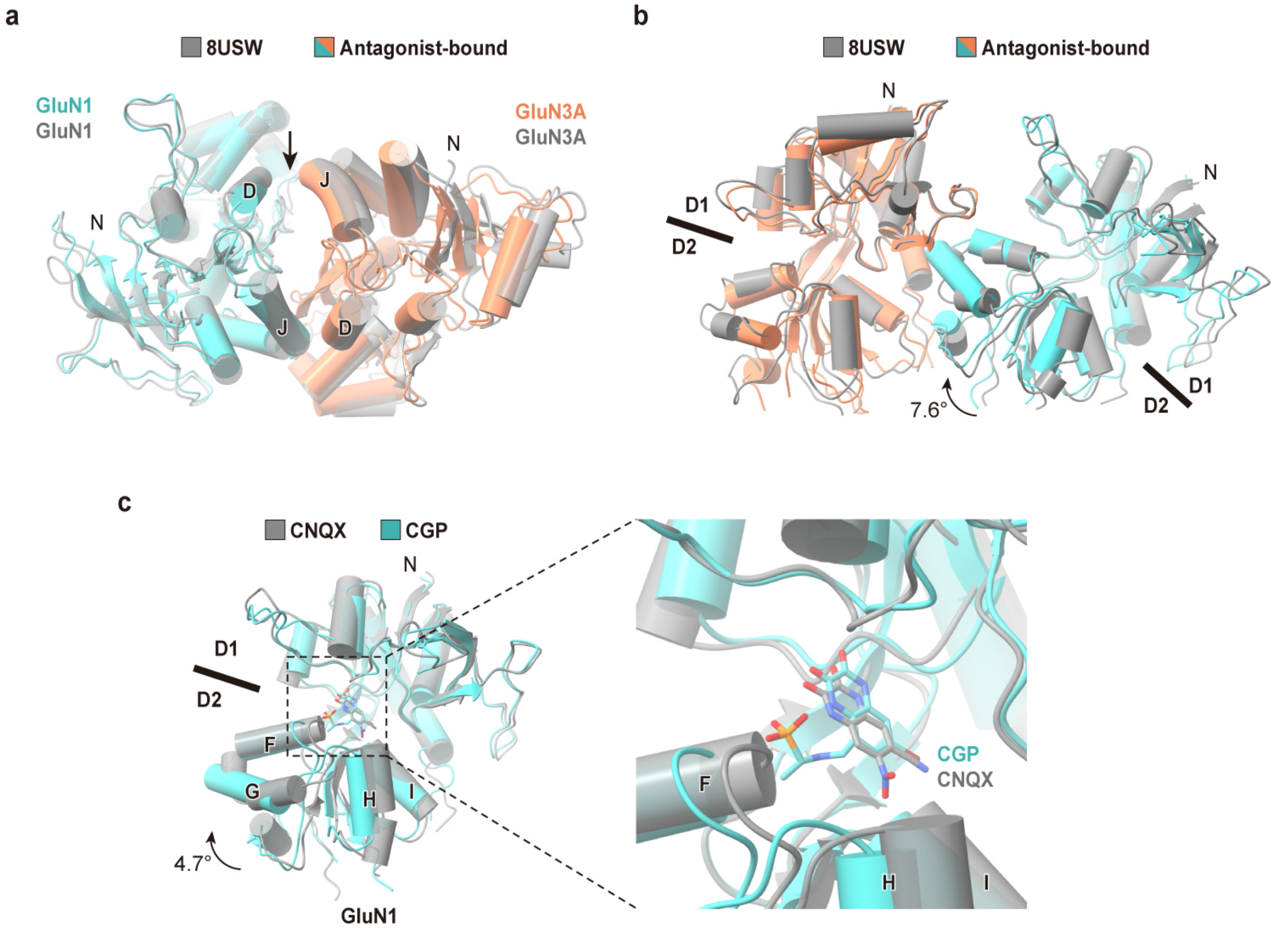

Supplementary Fig. 2: Structural comparison of GluN1-GluN3A D1-D1 interface between CGP- and CNQX-bound states. a, Superposition of GluN1-GluN3A LBD dimer structures in the CGP-bound (colored) and CNQX-bound (gray, PDB 8USW) states, aligned using the Cα atoms of the GluN1 D1 residues and viewed from the top. The D1-D1 interface is more extensive in the CGP-bound state with a contact area of 779 Å^2 (652 Å^2 in the CNQX-bound state), particularly at GluN1 helix D and GluN3A helix J (arrow). **b**, Comparison of the LBD dimer structures reveals that the D2 lobe of CGP-bound GluN1 rotates 7.6° toward the GluN3A LBD compared to the CNQX-bound GluN1. **c**, Superposition of GluN1 clamshells aligned at D1 reveals that the D2 lobe of CGP-bound GluN1 is twisted by 4.7° compared to the CNQX-bound D2 lobe. The GluN1 clamshell clefts show that CGP occupies larger space than CNQX with a branch and a phosphonate group (Right).

Supplementary Table 1. Functional properties for WT and mutant GluN1/GluN3A receptors*.

In the presence of 1 μM CGP

**10 ms exposure**	rise time (ms)	Desensitization τ_D (ms)	Desensitization % desens (I_{SS}/I_{peak})	Deactivation τ_{fast} (ms)	Deactivation τ_{slow} (ms)	Deactivation % fast	Deactivation τ_w (ms)	n
GluN1-4a/3A	6.9 ± 0.4	-	-	65 ± 8	424 ± 110	66 ± 9	122 ± 8	13
GluN1$_{EM}$/3A$_{EM}$	3.4 ± 0.1	-	-	14 ± 1	164 ± 12	87 ± 1	31 ± 1	15
GluN1-4a/3A-E889L+S892L	15 ± 1.3	-	-	603 ± 112	2090 ± 180	32 ± 11	1560 ± 80	9
GluN1$_{EM}$/3A$_{EM}$-E889L+S892L	5.6 ± 0.4	-	-	93 ± 12	886 ± 174	75 ± 6	267 ± 39	6

**2 s exposure**	rise time (ms)	τ_D (ms)	% desens (I_{SS}/I_{peak})	τ_{fast} (ms)	τ_{slow} (ms)	% fast	τ_w (ms)	n
GluN1-4a/3A	8.6 ± 0.6	53 ± 7	48 ± 3	129 ± 11	1060 ± 170	79 ± 6	272 ± 33	13
GluN1$_{EM}$/3A$_{EM}$	3.4 ± 0.1	16 ± 1	69 ± 3	56 ± 5	609 ± 68	72 ± 2	205 ± 19	13
GluN1-4a/3A-E889L+S892L	251 ± 62	-	-	539 ± 122	2240 ± 280	47 ± 10	1390 ± 130	8
GluN1$_{EM}$/3A$_{EM}$-E889L+S892L	163 ± 27	-	-	108 ± 7	1840 ± 270	79 ± 3	458 ± 51	6

**Concentration-response data**	Glycine EC_{50} (μM)	n_H	n
GluN1-4a/3A	92 ± 5	1.3	9
GluN1-4a/3A-E889L	19 ± 1	1.4	7
GluN1-4a/3A-S892L	3.4 ± 0.6	1.5	5
GluN1-4a/3A-E889L+S892L	1.1 ± 0.1	1.5	7
GluN1$_{EM}$/3A$_{EM}$	51 ± 9	1.2	8
GluN1$_{EM}$/3A$_{EM}$-E889L+S892L	9.7 ± 1.8	0.9	9

In the absence of CGP

**5 ms exposure**	rise time (ms)	Deactivation τ_{fast} (ms)	Deactivation τ_{slow} (ms)	Deactivation % fast	Deactivation τ_w (ms)	n
GluN1-4a/3A	3.0 ± 0.2	30 ± 9	260 ± 90	74 ± 4	67 ± 12	9
GluN1-4a/3A-E889L	3.8 ± 0.3	42 ± 9	304 ± 43	64 ± 4	132 ± 13	6
GluN1-4a/3A-S892L	3.6 ± 0.2	376 ± 60	1690 ± 140	64 ± 9	821 ± 81	6
GluN1-4a/3A-E889L+S892L	4.2 ± 0.1	393 ± 60	1920 ± 350	55 ± 11	903 ± 72	8

**3 s exposure**	rise time (ms)	Desensitization τ_{fast} (ms)	Desensitization τ_{slow} (ms)	Desensitization % fast	Desensitization $\tau_{D,w}$ (ms)	Desensitization % desens ($1-I_{SS}/I_{peak}$)	n
GluN1-4a/3A	2.9 ± 0.2	15 ± 2	133 ± 15	82 ± 4	34 ± 4	94 ± 1	16
GluN1-4a/3A-E889L	3.5 ± 0.2	28 ± 2	242 ± 20	85 ± 2	63 ± 6	97 ± 1	13
GluN1-4a/3A-S892L	3.8 ± 0.2	29 ± 3	338 ± 32	84 ± 3	77 ± 8	95 ± 1	11
GluN1-4a/3A-E889L+S892L	4.3 ± 0.1	61 ± 4	312 ± 29	82 ± 2	102 ± 8	99 ± 1	13

*Concentration-response data for glycine measured at steady-state in presence of 1 μM CGP and kinetic parameters describing macroscopic responses to glycine in the absence or presence of 1 μM CGP were determined for WT and mutant receptors expressed in HEK cells using whole-cell patch-clamp electrophysiology. Responses for the determination of kinetic parameters were activated by short (5 ms or 10 ms) or long (2 s or 3 s) exposures to 10 mM glycine. See Methods for details on data analysis. Data are shown as mean ± S.E.M. and n is the number of cells used in the determination.

Supplementary Table 2. Statistics of cryo-EM data collection and model refinement.

	Antagonist-bound GluN1$_{EM}$/GluN3A$_{EM}$			Activated GluN1$_{EM}$/GluN3A$^{ELSL}_{EM}$					Desensitized GluN1$_{EM}$/GluN3A$_{EM}$		
				Class 1, pre-active		Class 2, active		Class 3, open			
	LBD-TMD<br>(EMD-70399)<br>(PDB 9OEH)	LBD<br>(EMD-70433)<br>(PDB 9OFI)	TMD<br>(EMD-70446)<br>(PDB 9OFY)	LBD-TMD<br>(EMD-70402)<br>(PDB 9OEM)		LBD<br>(EMD-70403)<br>(PDB 9OEN)		LBD-TMD<br>(EMD-70404)	LBD-TMD<br>(EMD-70405)<br>(PDB 9OEO)	LBD<br>(EMD-70437)<br>(PDB 9OFM)	TMD<br>(EMD-70447)<br>(PDB 9OFZ)
**Data collection and processing**											
Ligands		CGP/DCKA		CGP/Gly/GNE-9278/UCM-A129						Gly	
Microscope		FEI Titan Krios		FEI Titan Krios				FEI Titan Krios		FEI Titan Krios	
Camera, energy filter		K3, BioContinuum		Falcon 4i, Selectris X				Falcon 4i, Selectris X		K3, BioContinuum	
Voltage (kV)		300		300				300		300	
Magnification		81,000		130,000				165,000		105,000	
Energy filter slit width (eV)		20		6				20		20	
Dose rate (e^-/pix/s)		8.5		7.6				8.9		16	
Total electron exposure (e^-/Å^2)		50		60				50		50	
Number of movie frames		60		2,115				1,096		46	
Pixel size (Å/pix)		0.8442		0.940				0.743		0.829	
Defocus range (μm)		-0.8 – -2.2		-0.8 – -2.2				-0.8 – -2.0		-1.0 – -2.4	
Number of micrographs		14,481		21,673				7,622		14,848	
Number of processed particles		80,690		60,645		311,684		14,421		173,386	
Symmetry imposed	*C*1	*C*1	*C*2	*C1*	*C*2	*C1*	*C*2	*C*1	*C*1	*C*1	*C*2
B-factor (Å^2)	92.5	86.3	113.8	69.4	84.4	81.2	88.2	70.5	166.4	156.3	144.7
Map resolution (Å), FSC=0.143	3.67	3.41	4.12	3.01	2.88	2.81	2.71	4.06	4.21	4.04	4.51
**Model refinement and validation**											
Model resolution (Å), FSC=0.5	3.9	3.7	4.6	3.3		2.9			4.5	4.3	5.1
Model composition											
Atoms	11,490	8,745	2,658	13,512		9,162			10,884	8,308	2,667
Protein residues	1,591	1,134	428	1,715		1,140			1,563	1,128	449
Ligands	12	12	0	20		14			10	10	0
Bond (RMSD)											
Length (Å)	0.003	0.003	0.002	0.003		0.006			0.003	0.002	0.003
Angle (°)	0.523	0.482	0.433	0.512		0.607			0.500	0.467	0.491
Validation											
MolProbity score	1.82	1.81	1.41	1.56		1.29			2.03	1.98	1.64
Clashscore	9.26	10.91	3.90	5.89		3.17			13.59	12.41	7.39
Ramachandran plot											
Outliers (%)	0.00	0.00	0.00	0.00		0.00			0.00	0.00	0.00
Allowed (%)	4.65	3.78	3.54	3.54		3.04			5.80	5.55	3.60
Favored (%)	95.35	96.22	96.46	96.46		96.96			94.20	94.45	96.40
Rotamer outliers (%)	0.30	0.92	0.74	0.60		0.41			0.35	0.80	0.00
C-beta outliers (%)	0.00	0.00	0.00	0.00		0.00			0.00	0.00	0.00
CC (model vs. map, mask)	0.81	0.80	0.76	0.82		0.85			0.76	0.76	0.76

Supplementary Table 3. Glycine potencies at WT and mutant GluN1/GluN3A receptors.

	Glycine EC_{50} (μM)	n_H	n
GluN1-4a/3A	26 ± 1	1.7	11
GluN1-4a-T550A/3A	29 ± 1	1.7	10
GluN1-4a-D552A/3A	27 ± 1	1.6	9
GluN1-4a-F554A/3A	30 ± 1	1.6	9
GluN1-4a-M555A/3A	24 ± 1	1.6	10
GluN1-4a-M555W/3A	25 ± 2	1.5	10

Concentration-response data for glycine were measured for WT and mutant receptors expressed in *Xenopus* oocytes using two-electrode voltage-clamp electrophysiology. The responses to increasing glycine concentrations were measured in the continuous presence of 1 μM CGP to prevent desensitization. EC_{50} values are shown as mean ± S.E.M., n_H is the Hill slope, and n is the number of oocytes used in the determination.
