# Supplementary material for: Structural and mechanistic insights into gating and allosteric modulation of GluN1–GluN3A NMDA receptors
Source: Nat Struct Mol Biol. 2026 Aug 28;33(9):1299–310. doi: 10.1038/s41594-026-01866-9 (PMC13561882; doi:10.1038/s41594-026-01866-9)
Supplement: Supplementary file 3 [file 41594_2026_1866_MOESM3_ESM.pdf]

nature portfolio

Peer Review File

Structural and mechanistic insights into gating and allosteric modulation of GluN1/GluN3A NMDA receptors

Corresponding Author: Dr Eric Gouaux

Version 0:

Decision Letter:

19th Dec 2025

Dear Dr. Gouaux,

Thank you again for submitting your manuscript "Native architecture, allosteric modulation and gating mechanism of glycine-dependent NMDA receptors". I apologize for the delay in sharing our decision. We have decided to send your manuscript for peer review, but several points require your attention before we can proceed with peer review.

I am re-opening the manuscript submission system for you to resubmit your manuscript with all associated files needed for peer review directly, within a week. Please follow the link at the bottom of this email to upload the documents listed below. If you have any issues, please reach out to us before completing the submission.

1. STRUCTURAL DATA:
We require official wwPDB validation reports for newly described atomic structures per journal policy. We also request that authors provide cryo-EM maps, half-maps and models, as well as maps and models obtained from subtomogram averaging if it applies to the work, to help the reviewers in assessing the work. We recommend the use of figshare in our system, which allows for provision of anonymous access links for the referees (https://www.springernature.com/gp/authors/research-data/figshare-integration). Alternatively, please upload .zip folders directly with the submission.
Manuscripts reporting new structures should contain a table summarizing structural and refinement statistics. Please include these tables for new cryo-EM or -ET (https://www.nature.com/documents/nr-tables-cryo-em.doc and modifying this file for ET), NMR (https://www.nature.com/documents/nr-tables-nmr.doc) and X-ray crystallography (https://www.nature.com/documents/nr-tables-xray.doc). To facilitate assessment of the quality of the structural data, a stereo image of a portion of the electron density map (for crystallography papers) or of the superimposed lowest energy structures (>10; for NMR papers) should be provided with the submitted manuscript. If the reported structure represents a novel overall fold, a stereo image of the entire structure (as a backbone trace) should be provided. For cryo-EM structures, a representative micrograph showing individual particles should be provided in the figures alongside a processing workflow (please see our Editorial for more information: https://www.nature.com/articles/s41594-025-01567-9 and here for an example: https://www.nature.com/articles/s41594-025-01586-6/figures/7).

2. REPORTING SUMMARY:
We want to ensure that the methods and statistics reporting in our papers are of the highest quality. To that end, we ask authors to fill out a Reporting Summary that collects information on experimental design and reagents. If your paper includes ChIP-seq or flow cytometry data, we ask that you take special care to complete those sections of the Reporting Summary as this data will aid greatly in the review of your manuscript.

Reporting Summary:
https://www.nature.com/documents/nr-reporting-summary.pdf

3- PROTEOMICS AND SEQUENCING:
Please provide accession numbers and reviewer tokens to access sequencing or proteomics datasets if any unpublished datasets are part of your study. Please add this information to your manuscript file. Proteomics data should be deposited to any ProteomeXchange member repository (http://www.proteomexchange.org/) and DNA sequencing, RNA sequencing, genome assembly data should be deposited to any INSDC member repository (https://www.insdc.org/) or Genome Sequence Archive.

4- CODE, MD SIMULATIONS, AND MACHINE LEARNING REPORTING:

Please provide the code used to analyse the data if newly developed (unpublished) code was used in the work. For the reviewers to evaluate the work adequately, they must be able to test the software/review the code themselves. If you have not yet provided the software, we therefore request that you provide a single compressed zip file containing the software with a readme.txt file or other user manual containing complete instructions for installing and running the software. If appropriate, please provide example data and expected output. Sufficient material should be provided for referees to directly test the performance of the software/algorithm. Alternatively, you can upload the code to GitHub and provide the link directly in the manuscript.

Please fill out and upload the code and software submission checklist that will be made available to editors and reviewers. This form is a dynamic 'smart pdf' and must therefore be downloaded and completed in Adobe Reader, instead of opening it in a web browser.
https://www.nature.com/documents/nr-software-policy.pdf

If molecular dynamics (MD) simulations were performed, please refer to this Editorial and provide a completed MD simulations checklist with your manuscript: https://www.nature.com/articles/s42003-023-04653-0

Lastly, if it applies, please complete the completed machine learning checklist with your submission and upload it for review: https://www.nature.com/documents/machine-learning-checklist.pdf

Please use the link below to submit the files. **Please also remember to move forward all other files associated with this version of the paper.**

Link Redacted

Note: This URL links to your confidential home page and associated information about manuscripts you may have submitted, or that you are reviewing for us. If you wish to forward this email to co-authors, please delete the link to your homepage.

We look forward to seeing the revised manuscript and thank you for the opportunity to review your work.

Sincerely,

Katarzyna Ciazynska, PhD
(she/her)
Senior Editor
Nature Structural & Molecular Biology
https://orcid.org/0000-0002-9899-2428

Version 1:

Decision Letter:

27th Feb 2026

Dear Dr Gouaux,

Thank you again for submitting your manuscript "Native architecture, allosteric modulation and gating mechanism of glycine-dependent NMDA receptors". Please accept my sincere apologies for the delay in this decision, which resulted from the difficulty in obtaining suitable referee reports.

We now have comments (below) from the 4 reviewers who evaluated your paper. In light of those reports, we remain interested in your study and would like to see your response to the comments of the referees in the form of a revised manuscript.

You will see that while reviewers appreciate the results, they raise several concerns which will need to be addressed in a revision. Specifically, in line with comments of reviewer #3, we ask that you provide further experimental support for pre-active and active conformations and their relation to wild-type receptor dynamics. Further, please provide more experimental support for the open state, in absence of reliable TMD density (point echoed by reviewer #4 as well). These points will be important to address for further editorial consideration. We also ask that you fully address comments regarding the stoichiometry analysis and photobleaching experiments, brought up be reviewers #1, #2 and #3, and discuss any caveats of the experimental system. Please provide needed controls for co-localisation experiments. We agree with reviewer #4 that additional structure of the antagonist bound the ELSL mutant could be informative, but we will not consider this point essential in the context of current work.

Our standard revision period is 4 months. We appreciate that the requested revisions are extensive. We are committed to providing a fair and constructive peer-review process and encourage you to contact us if you would like to discuss the reviews or anticipate any delay with the resubmission.

We will be happy to consider your revision as long as nothing similar has been accepted for publication at NSMB or published elsewhere. Should your manuscript be substantially delayed without notifying us in advance and your article is eventually published, the received date would be that of the revised, not the original, version.

REPORTING AND DATA AVAILABILITY: Please provide updated files as follows with your revision. As you already know, we put great emphasis on ensuring that the methods and statistics reported in our papers are correct and accurate.

1. REPORTING SUMMARY: Please provide an updated file:

https://www.nature.com/documents/nr-reporting-summary.pdf

This form is a dynamic 'smart pdf' and must be downloaded and completed in Adobe Reader.

2. REPORTING OF STRUCTURAL DATA: If there are additional or modified structures in the revision, please submit the corresponding PDB validation reports with your other files, and use the figshare integration system to provide access to maps, half-maps, and models.

Manuscripts reporting new structures should contain a table summarizing structural and refinement statistics. Please include these tables for new cryo-EM or -ET (https://www.nature.com/documents/nr-tables-cryo-em.doc and modifying this file for ET), NMR (https://www.nature.com/documents/nr-tables-nmr.doc) and X-ray crystallography (https://www.nature.com/documents/nr-tables-xray.doc). To facilitate assessment of the quality of the structural data, a stereo image of a portion of the electron density map (for crystallography papers) or of the superimposed lowest energy structures (>10; for NMR papers) should be provided with the submitted manuscript. If the reported structure represents a novel overall fold, a stereo image of the entire structure (as a backbone trace) should also be provided. For cryo-EM structures, a representative micrograph showing individual particles should be provided in the figures alongside a processing workflow (please see our Editorial for more information: https://www.nature.com/articles/s41594-025-01567-9).

DATA DEPOSITION: We require deposition of coordinates (and, in the case of crystal structures, structure factors) into the Protein Data Bank with the designation of immediate release upon publication (HPUB). Electron microscopy-derived density maps and coordinate data must be deposited in EMDB and released upon publication. Deposition and immediate release of NMR chemical shift assignments are requested. To avoid delays in publication, dataset accession numbers must be supplied with the revised manuscript and appropriate release dates must be indicated at the galley proof stage.

3. REPORTING OF LIGHT MICROSCOPY DATA: For any revision that includes light microscopy data, we ask our authors to please include a completed light microscopy reporting table [https://www.nature.com/documents/Light_microscopy_reporting_table.xlsx] to ensure the methods are described thoroughly. The table will be available to reviewers and ultimately published, should the manuscript be accepted at the journal. When submitting the revised version of your manuscript, please pay close attention to our href="https://www.nature.com/nature-portfolio/editorial-policies/image-integrity">Digital Image Integrity Guidelines.

4. CODE AND COMPUTATIONAL WORK: If newly developed, unpublished code was used in this work, it must be provided with the revision and access to it must be disclosed in the Code Availability Statement in the manuscript. Please also provide the completed software submission checklist. This form is a dynamic 'smart pdf' and must be downloaded and completed in Adobe Reader, instead of opening it in a web browser: https://www.nature.com/documents/nr-software-policy.pdf

If molecular dynamics (MD) simulations were performed, please refer to this Editorial and provide a completed MD simulations checklist with your revision: https://www.nature.com/articles/s42003-023-04653-0

Lastly, if it applies, please complete and upload the completed machine learning checklist with your submission for re-review: https://www.nature.com/documents/machine-learning-checklist.pdf

5. SOURCE GEL IMAGES: Unprocessed scans should be provided for all gels and western blots presented in figures and should be clearly labelled with the figure number in the file title. The source gel images should be uncropped, unmodified images, with molecular weight markers. Uncropped, unmodified gel images from existing experimental repeats, in addition to the data shown in the figures, can also be presented in source data. Please provide these files as PDF with your revision.

Please ensure that all control panels for gels and western blots are appropriately described as loading or IP controls. Lastly, please ensure that all images in the paper are checked for duplication of panels and for splicing of gel lanes.

6. SOURCE NUMERICAL DATA: we urge authors to provide, in tabular form in excel files, the data underlying the graphical representations used in figures. This is to further increase transparency in data reporting, as detailed in this editorial

(http://www.nature.com/nsmb/journal/v22/n10/full/nsmb.3110.html). Please provide one excel file per figure max, with one panel per tab. When submitting files, the title field should indicate which figure the source data pertains to.

7. DATA AVAILABILITY: this journal strongly supports public availability of data. All data used in accepted papers should be available via a public data repository, or alternatively, as Supplementary Information. If data can only be shared on request, please explain why in your Data Availability Statement and in the correspondence with your editor.

For some data types, deposition in a public repository is mandatory as detailed below:

https://www.nature.com/nature-research/editorial-policies/reporting-standards#availability-of-data

Finally, please ensure that you retain unprocessed data and metadata files after publication, ideally archiving data in perpetuity, as these may be requested during the peer review and production process or after publication if any issues arise.

EXTENDED DATA FIGURES

When re-submitting your manuscript, please ensure that any supplementary figures and tables that are crucial to the manuscript's conclusions are converted into Extended Data figures and tables to increase visibility of these data. Extended Data figures and tables are online-only (present in the online PDF and full-text HTML versions of the paper), peer-reviewed display items that provide essential background to the article but are not included in the main article due to space constraints. A maximum of ten Extended Data display items (figures and tables) is permitted.

While we encourage the use of color in preparing figures, please note that this will incur a charge to partially defray the cost of printing. Information about color charges can be found at http://www.nature.com/nsmb/authors/submit/index.html#costs

Nature Structural & Molecular Biology is committed to improving transparency in authorship. As part of our efforts in this direction, we are now requesting that all authors identified as 'corresponding author' on published papers create and link their Open Researcher and Contributor Identifier (ORCID) with their account on the Manuscript Tracking System (MTS), prior to acceptance. This applies to primary research papers only. ORCID helps the scientific community achieve unambiguous attribution of all scholarly contributions. You can create and link your ORCID from the home page of the MTS by clicking on 'Modify my Springer Nature account'. For more information please visit please visit www.springernature.com/orcid.

Please use the link below to submit your revised manuscript and related files:

Link Redacted

Note: This URL links to your confidential home page and associated information about manuscripts you may have submitted, or that you are reviewing for us. If you wish to forward this email to co-authors, please delete the link to your homepage.

We look forward to seeing the revised manuscript and thank you for the opportunity to review your work.

Sincerely,

Katarzyna Ciazynska, PhD
(she/her)
Senior Editor
Nature Structural & Molecular Biology
https://orcid.org/0000-0002-9899-2428

Referee expertise:

Referee #1: neurotransmission, NMDA receptors, molecular biology and biophysics

Referee #2: biophysics, membrane protein stoichiometry

Referee #3: cryo-EM, membrane proteins

Referee #4: NMDA receptors

Reviewers' Comments:

Reviewer #1 (Remarks to the Author):

The paper by Kim et al studies the full length GluN3A NMDA receptor. This receptor is unusual for responding to glycine alone and for “paradoxical” gating, whereby the N1 subunit inhibits current. The authors isolate native receptors and through single subunit counting with GFP photobleaching conclude that the native N3A has a 2:2 stoichiometry with N1. Through mutagenesis that identifies interactions that stabilize the elusive active state, a combination of orthosteric and allosteric ligands and electrophysiology and cryoEM the authors obtain structures of the major states of the receptor: resting; pre-active, active and desensitized.

Two important findings are particularly worth mentioning. First, activation is shown to depend on closure of N3 on glycine with little movement of N1, this prevents N3 from rotating and so prevents desensitization, explaining why antagonist-jammed N1 LBD “blocked-open” state prevents its movement and allows the channel to open. This is supported by the finding that the open gate is 2-fold symmetric, unlike the 4-fold symmetry of GluN2 receptors, in which all 4 subunits rearrange to open the channel. Second, the mechanism of inhibition by GluN1 is a transition from 2-fold symmetry to 4-fold symmetry when the N1 LBD closes on glycine, N1 rotates and then permits closed N3 to rotate.

The data quality is high, the presentation/logic and interpretations are well reasoned. The paper jumps around a bit with findings presented later in the paper alluded to in advance to explain approach or interpretation.

The paper adds substantially to the field, in obtaining higher resolution without cross-linking than before, successfully resolving the LBD-TMD linkers, the TMD and ion channel pore and determining pre-active and and active conformations as well as desensitization mechanisms.

The paper is highly deserving of publication. Just a few suggestions for clarity.

Specific Comments

Figure 1. Photobleaching. “Photobleaching analysis of the GFP signal revealed 105 that approximately 67% of the fluorophores bleach in two steps, while about 33% bleach in one 106 step (Fig. 1d,e and Extended Data Fig. 2c-e)”

Be explicit that GFP bleaching shows ~55% 2-step bleach and that this is interpreted as 67%, assuming that 20% of GFPs are not fluorescent.

Also, extended Fig images are too dim.

Figure 1. Co-localization. “capture GluN3A-containing receptors and probing with 5F11 [for N1] and another 109 GluN3A-specific antibody, 2D343, ~81% of GluN3A signals colocalized with GluN1 signals (Fig. 110 1f and Extended Data Fig. 2f)”

1f shows # of molecules, not clear how this is converted to 81%.
What is the control?

Figure 2. Indicate in 2d structures that correspond to classes 1,2 and 3.

Comparison of N3A to N2B receptors is made in Figures 5 and 6 and associated text. But the differences are mentioned earlier in the text without the clear documentation. This makes the text jumpy and a bit repetitive. It would be good to consolidate for clarity. For example, this appears in the section describing Figure 3, but without explanation: “We also speculate that the narrower cross-section of the GluN1/GluN3A receptor pore in the A/C direction, together with the positively charged arginine lining the GluN3A pore, could explain its low channel conductance.” Later it is shown and discussed in detail in Figures 5 and 6. Why not wait to mention it then?

Reviewer #2 (Remarks to the Author):

NMDA receptors (NMDAR) play central roles in synaptic plasticity and memory. NMDAR are heterotetramers that contain two copies of GluN1 subunits . We have a good structural understanding of GluN1/GluN2 NMDAR. However, only incomplete structures are available for the glycine-dependent NMDA receptor GluN1/Glu3A NMDAR. As a result, we have a poor understanding of several key aspects of these receptors. The manuscript by Kim et al. constitutes a significant advancement towards a mechanistic understanding of glycine-dependent NMDA receptor, as cryoEM structures for GluN1/Glu3A NMDAR in several functional states are provided. The structural wealth provided allows to rationalize how glycine activates NMDAR and how a GluN1 selective antagonist potentiates GluN3 activity.
The 2/2 stoichiometry of GluN1/Glu3A NMDAR is supported by a recent paper (Michalski, Science Advances 2024). However, in this work the transmembrane helices were crosslinked with disulfides, and therefore do not represent native

conditions. Therefore, it is important to use an unbiased assay to assess stoichiometry, as the manuscript does. The authors purify GluN1/Glu3A NMDAR from mouse brain, and perform single-molecule pull down to study stoichiometry from photobleaching steps. While the cryoEM section of the manuscript is compelling, some question remain about these studies that I would need addressed to be convinced:
-What photobleaching is expected for a 2/2 stoichiometry? If the binomial distribution is meant to be answering this question, that should be stated clearly. If so, clear differences between the data and the binomial distribution are found, that should be discussed. Can references be cited for similar assays? The dark state of GFP is a key consideration, but no references are provided for the estimated GFP maturation of 80%, which is higher than the value found in some references. One possibility
-Co-localization. Figure 1f is poorly described, and should be clearly explained. What are the two controls in the figure? The typo in the vertical axis should be fixed. How is the % of co-localization (81%) estimated? Looking at the image provided in Fig. Ext. 2f, I would not reach that conclusion.

The SiMPull data was obtained from mouse brain, while the cryoEM was expressed in HEK293T/17 cells, which are two different cellular context that might affect the stoichiometry. These differences should be clearly acknowledged as potential limitations of this study.
Minor comment:
-In figure 7, it is not obvious how the molecule GNE-9278 fits into the density. Could the authors fit into an Extended figure?
Overall, this manuscript makes important contributions to the field. The manuscript is written in a clear and concise prose, and is enjoyable to read.

Reviewer #3 (Remarks to the Author):

In this manuscript, Kim et al. investigate the native stoichiometry, structural architecture, gating transitions, desensitization mechanism, and pharmacological modulation of glycine-activated GluN1/GluN3A NMDA receptors. Using single-molecule pulldown (SiMPull) analysis, the authors demonstrate that native GluN3A-containing receptors in neonatal mouse brain predominantly assemble as diheteromeric GluN1/GluN3A tetramers. They then determine multiple cryo-EM structures of recombinant GluN1/GluN3A receptors in antagonist-bound, pre-active, active, and desensitized states, achieving resolutions sufficient to model LBD–TMD linkers and the ion channel pore.

The structural data reveal that glycine binding to GluN3A subunits drives activation while GluN1 remains antagonist-bound in an open clamshell conformation when CGP-78608 is present. Channel opening proceeds with approximate two-fold symmetry, distinct from the four-fold symmetric gating of canonical GluN1/GluN2 receptors. In contrast, desensitization involves a dramatic pseudo four-fold symmetric rearrangement of the LBD layer, reminiscent of AMPA/kainate receptor desensitization. The authors further elucidate the structural basis of paradoxical potentiation by the GluN1-selective antagonist CGP and map binding sites for two positive allosteric modulators (GNE-9278 and UCM-A129).
Overall, the study provides a comprehensive structural and mechanistic framework for understanding GluN1/GluN3A receptor gating and modulation, significantly advancing the field of NMDA receptor biology. With respect to novelty, previous structures of GluN1/GluN3 were already reported, which utilized engineered disulfide-bonds by Michalski & Furukawa in 2024 - an analogous approach to the engineered interface required for structure determination in the current study. Nevertheless, some specific aspects of this manuscript show more details, compared to the previous study, including slightly improved resolution (although the TMDs of most structures are overall of rather poor quality), and the presence of the linker region between LBD and TMD (although most structures have poorly resolved linkers). In addition to these technical aspects, I have a series of additional reservations regarding the claims and conclusions within the manuscript.

Major points:
1. The active-state structure relies on the GluN3A E889L/S892L (ELSL) mutant, which stabilizes the LBD D1–D1 interface and markedly reduces desensitization. While electrophysiological data show preserved glycine potency and increased open probability, this engineered stabilization may alter native gating energetics and conformational coupling. As it stands, it remains unclear whether the observed pre-active and active conformations fully reflect wild-type receptor dynamics. The authors should more explicitly discuss how the ELSL mutation might alter the activation pathway, and ideally provide additional functional or structural, or at the very least molecular dynamics-based evidence that the observed open conformation is representative of the wild-type receptor.

2. The identification of class 3 as an “open” state is based on an entirely unresolved TMD density and inferred M3 displacement, but no full atomic model is presented. The pore dimensions and ion conduction compatibility cannot be quantitatively validated (e.g., via pore radius analysis). Without electrophysiological correlation to specific structural states (e.g., via state-selective trapping or conductance measurements), assignment of the open state remains inferential. The absence of resolved selectivity filter conformational changes in the open state limits mechanistic certainty. The authors should provide additional structural, or MD simulation-based evidence that the observed state indeed adopts an “open” conformation. Finally, have the authors considered the possibility that this state is not, in fact, C2 symmetric, and the TMD is poorly resolved because C2 symmetry was applied?

3. The SiMPull data suggest that the predominant native assembly in neonatal mouse brain is diheteromeric GluN1/GluN3A. However:
Approximately one-third of complexes show single-step photobleaching. The study does not rule out the presence of triheteromeric GluN1/GluN2/GluN3A assemblies in other developmental stages or brain regions. Given the physiological

importance of triheteromeric assemblies reported in prior literature, the authors should more carefully delimit their conclusions to the specific developmental stage and preparation analyzed.

4. Antibody 5E3: The novel antibody 5E3, which specifically targets GluN3A is poorly described. How was it isolated/engineered and what is the epitope? Is it possible that the antibody skews the stoichiometry towards specific heteromers?

5. Naturally, a large emphasis of the paper is focused on symmetry of the respective reconstructions (2fold and 4fold), while very little information is provided about the exact details regarding processing with symmetry applied. Have the authors attempted C1 reconstructions for all maps? How confident are they that symmetry is indeed present and not just assumed?

6. The 'neural network'/cryoDRGN based analysis does not appear of very high quality. The movies provided show very low resolution reconstructions, and require lowering thresholds significantly, to see the respective densities. Ultimately the changes within these movies seem rather stochastic and densities appear and disappear, rather than showing clear conformational trajectories/deformations. Additional validation through other software packages (or established ones) would be critical to validate these findings.

Minor points:
1. What was the rationale for not running a local refinement on the 'pre-active' state structure? All other states appears to have separate refinements for LBD and TMD.

Reviewer #4 (Remarks to the Author):

This paper reports a substantial body of new information on NR3 subunit NMDA receptors, which paradoxically are activated by glycine and not glutamate. These receptors reach peak expression during early development, when they regulate synapse development, but their molecular structure and function is the least well understood amongst the NMDA receptor family. I anticipate that the study will be of interest to both the large community working on glutamate receptors and also to membrane protein structural biologists working on ion channels.

Comments

The key findings are

(1) The first definition, using single molecule pulldown and photobleaching, of a stoichiometry of 2NR1/2NR3A for native receptors purified from CNS tissue. The data clearly shows that this is the major species, with only a small population of GluN3A receptors with a potentially different stoichiometry. This 2:2 stoichiometry was validated in structural studies on recombinant receptor assemblies.

(2) Establishment of a structurally based gating scheme from the resting, to pre-active, to active, to desensitized states, achieved via cryo-EM analysis of multiple structures under conditions designed to trap these states using a combination of agonists and antagonists, mutants, and allosteric modulators. Notably this substantially extends our understanding of the gating cycle, which was not possible based on prior work, with the caveat that the ion channel structure in the active state is not well resolved.

(3) The first resolution of the ion channel structure (in the resting and pre-active states) including the pore helix and selectivity filter. This is a major step forwards and explains the low Ca permeability, and attenuated block by Mg that characterize NR3 activity, and the distinct V-gate that seals the pore in the closed state. Likewise, resolution of the LBD-TMD linker density reveals unique structures that underlie gating in NR1/NR3A assemblies, and how these are distinct from that in NR1/NR2B. All of these features, i.e. both pore and linker structures, were unresolved in prior work by Michalski & Furukawa.

(4) Identification of novel binding sites for two allosteric modulators that stabilize the open state, opening the field for design of novel ligands.

(5) Resolution of a long-standing paradox for the mechanism by which the NR1 glycine site competitive antagonist "CGP" potentiates the activation of NR1/NR3A, with steric hindrance by the CGP bound NR1 LBD open cleft conformation preventing rotation of the LBD assembly into the 4-fold symmetric desensitized state previously found for kainate receptors.

The paper is well written and the experiments and analysis of exceptionally high quality. Of note, an impressive number of functional experiments are performed using receptors with structure informed mutations (e.g. Fig 7 and Extended data Figs 8 and 9). Overall, while comparison with the 2024 Michalski & Furukawa paper is inevitable, the much more comprehensive body of data reported here represents a landmark study that firmly puts our understanding of NR3 receptors on a solid basis. Discussion of prior work is appropriate and fair. Of interest, using a recently developed neural-network reconstruction process, it is shown that the ATD assembly of NR1/NR3A receptors is highly dynamic, sampling a continuum of conformations, preventing accurate structural analysis of this region, as confirmed by Q-score analysis of prior NR1/NR3A receptor structures, and the marginal quality of previously reported maps for this region.

In my opinion the paper tics all the boxes for NSMB.

Minor comments

Title: Because GluN1/GluN2 receptors are also glycine-dependent, perhaps change “glycine-dependent NMDA receptors”, to glycine-activated NMDA receptors?

Line 158: Rephrase ‘primarily distinguished by their TMD conformations’. This is a bit tricky given the incompletely resolved TMD density for the active state, and absence of a structural model to define an active conformation. Extended data Fig 6 compares raw electron density, but does not show structures. The more detailed discussion of the active state on pages 12-13 more accurately describes the data and its limitations.

Line 238: clarify if the 21.4° degree rotation refers to the lower lobe of the LBD, or does the whole GluN3A ELSL mutant LBD (including the upper lobe) rotate? If the latter case is true, is it possible to distinguish whether the conformational differences are in part due to the ELSL mutant versus the wild type antagonist bound structure, or to the binding of glycine to the GluN3A subunit? It would be useful to have a structure of the antagonist bound the ELSL mutant for comparison.

Line 274: the 4-fold symmetric structure for the kainate receptor desensitized state was first reported by Schauder et al 2013, PNAS PMID: 23530186 and should be cited here.

Line 289: the authors speculate, reasonably, that the ELSL mutant stabilizes the D1 dimer assembly; experimental support for this hypothesis is reported in Table 1 which reports a 4-fold slower time constant for desensitization the ELSL mutant compared to that for wild type GluN1/GluN3A. This should be reported in the main text.

Line 389: ‘remains unbound’. This needs justification or revision. 5,7-DCKA was present at 5 mM and would be anticipated to bind, but not with full occupancy, based on its published affinity of 650 µM for GluN3A; perhaps the absence of density might be due to partial occupancy? Was weak density observed with a different contour level?

Line 416: for clarity it would be useful to state: we cannot exclude the occurrence of minor populations of GluN3A receptors with different stoichiometries, but note that di-heteromeric GluN1/GluN3A receptors predominate.

Line 418 missing references (38-40) See also comment for line 274.

Line 719 FSEC was performed

Line 987 sharpen should be sharpened, line 991 A low pass filter, and line 995 unsharp should be unsharpened

Figure 2a legend: state the duration of the glycine applications for the left and right hand panels; same for panel c. Same for Extended data Fig. 8 d and h.

Supplementary Fig 1 panel C. The large difference in Q scores across the structures would be better emphasized if the Y-axis was plotted using the same scale for all panels, i.e. 0.8 to -0.6, with a dotted line at zero in all panels. Likewise, the X-axis should be from 0 to 950 in all panels, to fairly compare the Q-scores in the LBD segment.

Version 2:

Decision Letter:

Our ref: NSMB-A52208B

10th Jun 2026

Dear Dr. Gouaux,

Thank you for submitting your revised manuscript "Native architecture, allosteric modulation and gating mechanism of glycine-activated NMDA receptors" (NSMB-A52208B). It has now been seen by the original referees and their comments are below. The reviewers find that the paper has improved in revision, and therefore we'll be happy in principle to publish it in Nature Structural & Molecular Biology, pending minor revisions to satisfy the referees' final requests and to comply with our editorial and formatting guidelines.

We are now performing detailed checks on your paper and will send you a checklist detailing our editorial and formatting requirements in a couple of weeks. Please do not upload the final materials and make any revisions until you receive this additional information from us.

Thank you again for your interest in Nature Structural & Molecular Biology Please do not hesitate to contact me if you have any questions.

Sincerely,

Kat

Katarzyna Ciazynska, PhD
(she/her)
Senior Editor
Nature Structural & Molecular Biology
https://orcid.org/0000-0002-9899-2428

Reviewer #1 (Remarks to the Author):

The authors have done a good job of addressing the reviewer comments.
In the eyes of this reviewer, the paper is acceptable for publication.

Reviewer #2 (Remarks to the Author):

The authors have adequately addressed all the concerns I raised previously. The manuscript in its current form seems like an important addition to the bibliography.

Reviewer #3 (Remarks to the Author):

The authors have addressed all my major concerns.

Reviewer #4 (Remarks to the Author):

The revised manuscript addresses all of the issues raised by referees in the prior round of reviews, supplying new experimental data for assays of receptor function, and more detailed descriptions of cryo-EM data processing.

The study convincingly establishes the stoichiometry of assembly of native GluN3A receptors; provides the first structural evidence underlying the gating cycle of GluN3A receptors, and provides a structural explaination for why the highly selective GluN1 antagonist CGP potentiates GluN3A activity by blocking the conformational change triggered by binding of glycine to the GluN1 subunit that triggers desensitization. This is a substantial advance in our understanding of NMDA receptor biology.

The cryoDRGN analysis, with similar results obtained using other programs strongly supports the conclusion that the ATD domain is highly flexible, and I have no concerns with this analysis.

I support publication with no further revision.

INTERNAL USE ONLY AIP modules required: ["Clinical","Life Science","Chemistry"]

Version 3:

Decision Letter:

23rd Jul 2026

Dear Dr. Gouaux,

We are now happy to accept your revised paper "Structural and mechanistic insights into gating and allosteric modulation of GluN1/GluN3A NMDA receptors" for publication as an Article in Nature Structural & Molecular Biology.

Acceptance is conditional on the manuscript's not being published elsewhere and on there being no announcement of this work to the newspapers, magazines, radio or television until the publication date in Nature Structural & Molecular Biology.

Over the next few weeks, your paper will be copyedited to ensure that it conforms to Nature Structural & Molecular Biology style. Once your paper is typeset, you will receive an email with a link to choose the appropriate publishing options for your paper and our Author Services team will be in touch regarding any additional information that may be required.

After the grant of rights is completed, you will receive a link to your electronic proof via email with a request to make any

corrections within 48 hours. If, when you receive your proof, you cannot meet this deadline, please inform us at rjsproduction@springernature.com immediately.

You will not receive your proofs until the publishing agreement has been received through our system.

Due to the importance of these deadlines, we ask that you please let us know now whether you will be difficult to contact over the next month. If this is the case, we ask you provide us with the contact information (email, phone and fax) of someone who will be able to check the proofs on your behalf, and who will be available to address any last-minute problems.

To assist our authors in disseminating their research to the broader community, our SharedIt initiative provides all co-authors with the ability to generate a unique shareable link that will allow anyone (with or without a subscription) to read the published article. Recipients of the link with a subscription will also be able to download and print the PDF.

As soon as your article is published, you can generate your shareable link by entering the DOI of your article here: http://authors.springernature.com/share. Corresponding authors will also receive an automated email with the shareable link

Note the policy of the journal on data deposition: http://www.nature.com/authors/policies/availability.html.

Your paper will be published online soon after we receive proof corrections and will appear in print in the next available issue. You can find out your date of online publication by contacting the production team shortly after sending your proof corrections.

You may wish to make your media relations office aware of your accepted publication, in case they consider it appropriate to organize some internal or external publicity. Once your paper has been scheduled you will receive an email confirming the publication details. This is normally 3-4 working days in advance of publication. If you need additional notice of the date and time of publication, please let the production team know when you receive the proof of your article to ensure there is sufficient time to coordinate. Further information on our embargo policies can be found here: https://www.nature.com/authors/policies/embargo.html

You can now use a single sign-on for all your accounts, view the status of all your manuscript submissions and reviews, access usage statistics for your published articles and download a record of your refereeing activity for the Nature journals.

If you have not already done so, we strongly recommend that you upload the step-by-step protocols used in this manuscript to the Protocol Exchange. Protocol Exchange is an open online resource that allows researchers to share their detailed experimental know-how. All uploaded protocols are made freely available, assigned DOIs for ease of citation and fully searchable through nature.com. Protocols can be linked to any publications in which they are used and will be linked to from your article. You can also establish a dedicated page to collect all your lab Protocols. By uploading your Protocols to Protocol Exchange, you are enabling researchers to more readily reproduce or adapt the methodology you use, as well as increasing the visibility of your protocols and papers. Upload your Protocols at www.nature.com/protocolexchange/. Further information can be found at www.nature.com/protocolexchange/about.

An online order form for reprints of your paper is available at https://www.nature.com/reprints/author-reprints.html. Please let your coauthors and your institutions' public affairs office know that they are also welcome to order reprints by this method.

Authors may need to take specific actions to achieve compliance with funder and institutional open access mandates. If your research is supported by a funder that requires immediate open access (e.g. according to Plan S principles or the NIH public access policy) then you should select the gold OA route, and we will direct you to the compliant route where possible. Because authors warrant under our subscription licensing terms that they haven't committed to licensing any version of their article under a licence inconsistent with the terms of our agreement – including the applicable embargo period – publication under the subscription model isn't suitable for authors whose funders require no embargo.

In approximately 10 business days you will receive an email with a link to choose the appropriate publishing options for your paper and our Author Services team will be in touch regarding any additional information that may be required.

You will not receive your proofs until the publishing agreement has been received through our system.

If you have any questions about our publishing options, costs, Open Access requirements, or our legal forms, please contact ASJournals@springernature.com

Sincerely,

Katarzyna Ciazynska, PhD
(she/her)

Senior Editor
Nature Structural & Molecular Biology
https://orcid.org/0000-0002-9899-2428

Click here if you would like to recommend Nature Structural & Molecular Biology to your librarian:
http://www.nature.com/subscriptions/recommend.html#forms

Open Access This Peer Review File is licensed under a Creative Commons Attribution 4.0 International License, which permits use, sharing, adaptation, distribution and reproduction in any medium or format, as long as you give appropriate credit to the original author(s) and the source, provide a link to the Creative Commons license, and indicate if changes were made.
In cases where reviewers are anonymous, credit should be given to 'Anonymous Referee' and the source.
The images or other third party material in this Peer Review File are included in the article’s Creative Commons license, unless indicated otherwise in a credit line to the material. If material is not included in the article’s Creative Commons license and your intended use is not permitted by statutory regulation or exceeds the permitted use, you will need to obtain permission directly from the copyright holder.
To view a copy of this license, visit https://creativecommons.org/licenses/by/4.0/

Thank you for taking the time to review our manuscript. Below, in blue text, we respond to the reviewers' comments.

Reviewers' Comments:

Reviewer #1 (Remarks to the Author):

The paper by Kim et al studies the full length GluN3A NMDA receptor. This receptor is unusual for responding to glycine alone and for "paradoxical" gating, whereby the N1 subunit inhibits current. The authors isolate native receptors and through single subunit counting with GFP photobleaching conclude that the native N3A has a 2:2 stoichiometry with N1. Through mutagenesis that identifies interactions that stabilize the elusive active state, a combination of orthosteric and allosteric ligands and electrophysiology and cryoEM the authors obtain structures of the major states of the receptor: resting; pre-active, active and desensitized.

Two important findings are particularly worth mentioning. First, activation is shown to depend on closure of N3 on glycine with little movement of N1, this prevents N3 from rotating and so prevents desensitization, explaining why antagonist-jammed N1 LBD "blocked-open" state prevents its movement and allows the channel to open. This is supported by the finding that the open gate is 2-fold symmetric, unlike the 4-fold symmetry of GluN2 receptors, in which all 4 subunits rearrange to open the channel. Second, the mechanism of inhibition by GluN1 is a transition from 2-fold symmetry to 4-fold symmetry when the N1 LBD closes on glycine, N1 rotates and then permits closed N3 to rotate.

The data quality is high, the presentation/logic and interpretations are well reasoned. The paper jumps around a bit with findings presented later in the paper alluded to in advance to explain approach or interpretation.

The paper adds substantially to the field, in obtaining higher resolution without cross-linking than before, successfully resolving the LBD-TMD linkers, the TMD and ion channel pore and determining pre-active and and active conformations as well as desensitization mechanisms.

The paper is highly deserving of publication. Just a few suggestions for clarity.

Specific Comments

Figure 1. Photobleaching. "Photobleaching analysis of the GFP signal revealed 105 that approximately 67% of the fluorophores bleach in two steps, while about 33% bleach in one 106 step (Fig. 1d,e and Extended Data Fig. 2c-e)"

Be explicit that GFP bleaching shows ~55% 2-step bleach and that this is interpreted as 67%, assuming that 20% of GFPs are not fluorescent.

Also, extended Fig images are too dim.

We have revised the sentences in lines 105-108 to clarify this point. Now it reads "*Photobleaching analysis of the GFP signal revealed that ~53% of the fluorophores exhibited two-step bleaching. By fitting the bleaching data to a binomial distribution, and accounting for incomplete GFP maturation (~80%)[46], we estimate that ~67% of the complexes have 2 GluN3A subunits (Fig. 1d,e and Extended Data Fig. 2c-e)*". We also refer to the Methods section where a more detailed description of GFP maturation has been added (lines 765–766), which reads "*The binomial distribution was calculated assuming 80% GFP maturation, as determined from a previous study[46]*". We have also adjusted the SiMPull images in Extended Data Fig. 2 to improve visibility.

Figure 1. Co-localization. "capture GluN3A-containing receptors and probing with 5F11 [for N1] and another 109 GluN3A-specific antibody, 2D343, ~81% of GluN3A signals colocalized with GluN1 signals (Fig. 110 1f and Extended Data Fig. 2f)"

1f shows # of molecules, not clear how this is converted to 81%.
What is the control?

We have revised the sentence to clarify these point (lines 114-115), which now reads "*...~81% of GluN3A signals (2D3-Alexa647; 66 ± 5 out of 81 ± 7) colocalized with GluN1 signals (5F11-Alexa594)...*". The controls in the SiMPull experiment measure non-specific binding of fluorescently labeled antibodies to the glass surface. These controls were performed by applying the non-biotinylated 'capture' antibody to a separate chamber while all other steps remained identical. Details are described in the Methods section (lines 751-753), and the figure legend has been updated to refer to the methods.

Figure 2. Indicate in 2d structures that correspond to classes 1,2 and 3.

We have indicated the pre-active and active states as class 1 and 2, respectively, in the figure legend.

Comparison of N3A to N2B receptors is made in Figures 5 and 6 and associated text. But the differences are mentioned earlier in the text without the clear documentation. This makes the text jumpy and a bit repetitive. It would be good to consolidate for clarity. For example, this appears in the section describing Figure 3, but without explanation: "We also speculate that the narrower cross-section of the GluN1/GluN3A receptor pore in the A/C direction, together with the positively charged arginine lining the GluN3A pore, could explain its low channel conductance." Later it is shown and discussed in detail in Figures 5 and 6. Why not wait to mention it then?

We have moved this sentence to lines 330–332 in the revised manuscript within the section describing Fig. 5.

Reviewer #2 (Remarks to the Author):

NMDA receptors (NMDAR) play central roles in synaptic plasticity and memory. NMDAR are

heterotetramers that contain two copies of GluN1 subunits . We have a good structural understanding of GluN1/GluN2 NMDAR. However, only incomplete structures are available for the glycine-dependent NMDA receptor GluN1/Glu3A NMDAR. As a result, we have a poor understanding of several key aspects of these receptors. The manuscript by Kim et al. constitutes a significant advancement towards a mechanistic understanding of glycine-dependent NMDA receptor, as cryoEM structures for GluN1/Glu3A NMDAR in several functional states are provided. The structural wealth provided allows to rationalize how glycine activates NMDAR and how a GluN1 selective antagonist potentiates GluN3 activity.
The 2/2 stoichiometry of GluN1/Glu3A NMDAR is supported by a recent paper (Michalski, Science Advances 2024). However, in this work the transmembrane helices were crosslinked with disulfides, and therefore do not represent native conditions. Therefore, it is important to use an unbiased assay to assess stoichiometry, as the manuscript does. The authors purify GluN1/Glu3A NMDAR from mouse brain, and perform single-molecule pull down to study stoichiometry from photobleaching steps. While the cryoEM section of the manuscript is compelling, some question remain about these studies that I would need addressed to be convinced:
-What photobleaching is expected for a 2/2 stoichiometry? If the binomial distribution is meant to be answering this question, that should be stated clearly. If so, clear differences between the data and the binomial distribution are found, that should be discussed. Can references be cited for similar assays? The dark state of GFP is a key consideration, but no references are provided for the estimated GFP maturation of 80%, which is higher than the value found in some references. One possibility

Yes we used binomial distribution to describe the 2:2 GluN1/GluN3A stoichiometry. We have clarified in the revised manuscript the differences between the observed data and binomial estimates (lines 105-108), which reads "*Photobleaching analysis of the GFP signal revealed that ~53% of the fluorophores exhibited two-step bleaching. By fitting the bleaching data to a binomial distribution, and accounting for incomplete GFP maturation (~80%)[46], we estimate that ~67% of the complexes have 2 GluN3A subunits (Fig. 1d,e and Extended Data Fig. 2c-e)*". We also have added a citation (ref.46) for the estimated GFP maturation of 80% in the revised manuscript. Although a direct estimate of GFP maturation efficiency for secreted Fab–GFP produced in insect cells is not available, a previous study using a similar single-molecule photobleaching assay reported a maturation efficiency of 79.5% for monomeric EGFP, the same fluorophore used in this study. We therefore used this value as an approximation in our analysis and have added this statement to the Methods section (lines 765–766), which reads "*The binomial distribution was calculated assuming an 80% GFP maturation, as determined in a previous study*[46]".

-Co-localization. Figure 1f is poorly described, and should be clearly explained. What are the two controls in the figure? The typo in the vertical axis should be fixed. How is the % of co-localization (81%) estimated? Looking at the image provided in Fig. Ext. 2f, I would not reach that conclusion.

The controls in the SiMPull experiment measure non-specific binding of fluorescence-labeled antibodies to the glass surface. These controls were performed by applying the non-biotinylated 'capture' antibody to a separate chamber while all other steps remained identical. Details are described in the Methods section (lines 751-753), and the figure legend has been updated to refer to it. Thank you for pointing out the typo, it has now been corrected. The 81% co-localization was

calculated as the fraction of GluN3A signals (2D3-Alexa647, 66 ± 5 out of 81 ± 7) that co-localize with GluN1 signals (5F11-Alexa594), i.e. 66/81 = 0.81. We have revised the sentence in lines 114-115 to state this point.

The SiMPull data was obtained from mouse brain, while the cryoEM was expressed in HEK293T/17 cells, which are two different cellular context that might affect the stoichiometry. These differences should be clearly acknowledged as potential limitations of this study.

We have now acknowledged this point in the revised manuscript lines 439-444, which read "*We determined the GluN1/GluN3A stoichiometry from mouse brain using SiMPull analysis, whereas the cryo-EM structures were obtained from recombinant receptors… Differences in cellular context and the effects of antibody binding may influence receptor assembly and the observed stoichiometry, both of which are limitations of this study*".

Minor comment:
-In figure 7, it is not obvious how the molecule GNE-9278 fits into the density. Could the authors fit into an Extended figure?

We have revised the Extended Data Fig.10b to show how the GNE molecule fits into the density map.

Overall, this manuscript makes important contributions to the field. The manuscript is written in a clear and concise prose, and is enjoyable to read.

Reviewer #3 (Remarks to the Author):

In this manuscript, Kim et al. investigate the native stoichiometry, structural architecture, gating transitions, desensitization mechanism, and pharmacological modulation of glycine-activated GluN1/GluN3A NMDA receptors. Using single-molecule pulldown (SiMPull) analysis, the authors demonstrate that native GluN3A-containing receptors in neonatal mouse brain predominantly assemble as diheteromeric GluN1/GluN3A tetramers. They then determine multiple cryo-EM structures of recombinant GluN1/GluN3A receptors in antagonist-bound, pre-active, active, and desensitized states, achieving resolutions sufficient to model LBD–TMD linkers and the ion channel pore.

The structural data reveal that glycine binding to GluN3A subunits drives activation while GluN1 remains antagonist-bound in an open clamshell conformation when CGP-78608 is present. Channel opening proceeds with approximate two-fold symmetry, distinct from the four-fold symmetric gating of canonical GluN1/GluN2 receptors. In contrast, desensitization involves a dramatic pseudo four-fold symmetric rearrangement of the LBD layer, reminiscent of AMPA/kainate receptor desensitization. The authors further elucidate the structural basis of paradoxical potentiation by the GluN1-selective antagonist CGP and map binding sites for two positive allosteric modulators (GNE-9278 and UCM-A129).
Overall, the study provides a comprehensive structural and mechanistic framework for understanding GluN1/GluN3A receptor gating and modulation, significantly advancing the field of

NMDA receptor biology. With respect to novelty, previous structures of GluN1/GluN3 were already reported, which utilized engineered disulfide-bonds by Michalski & Furukawa in 2024 - an analogous approach to the engineered interface required for structure determination in the current study. Nevertheless, some specific aspects of this manuscript show more details, compared to the previous study, including slightly improved resolution (although the TMDs of most structures are overall of rather poor quality), and the presence of the linker region between LBD and TMD (although most structures have poorly resolved linkers). In addition to these technical aspects, I have a series of additional reservations regarding the claims and conclusions within the manuscript.

Major points:

1. The active-state structure relies on the GluN3A E889L/S892L (ELSL) mutant, which stabilizes the LBD D1–D1 interface and markedly reduces desensitization. While electrophysiological data show preserved glycine potency and increased open probability, this engineered stabilization may alter native gating energetics and conformational coupling. As it stands, it remains unclear whether the observed pre-active and active conformations fully reflect wild-type receptor dynamics. The authors should more explicitly discuss how the ELSL mutation might alter the activation pathway, and ideally provide additional functional or structural, or at the very least molecular dynamics-based evidence that the observed open conformation is representative of the wild-type receptor.

We appreciate these comments. We acknowledge that the kinetics of the ELSL mutant shown in Supplementary Table 1 were not clearly described in the main text of the previous manuscript. In the revised manuscript, we have included additional electrophysiological recordings showing that the ELSL mutant undergoes nearly complete desensitization upon glycine application in the absence of CGP, similar to the wild-type receptor. Given the site of the substitutions, we expect to see changes in the function of the mutant receptor, especially related to entry into and recovery from desensitization. Most importantly, however, the electrophysiology data support the conclusion that the mutant receptor visits functionally relevant states visualized in our cryo-EM studies. In addition, we performed unitary conductance measurements for both mutant and wild-type receptors (see below), which show that their conductances are similar, although statistically different. Because simple pore models suggest that conductance is proportional to the cross-sectional area of the pore ($\propto r^2$), the measured conductances correspond to a difference in pore radius of only ~0.45 Å. These results therefore suggest that the pore dimensions, or cross-sectional areas, of the mutant receptor are similar to the wild-type parent and thus are consistent with the speculation that the open state conformations are also similar.

Together, our data indicate that the ELSL mutant preserves key features of wild-type receptor functional states, supporting the interpretation that the pre-active and active conformations reflect physiologically relevant conformations. We have revised the main text (lines 152–163) to clarify these points, which now reads "*Moreover, GluN1/GluN3A^{ELSL} receptors exhibit increased glycine potency compared to WT receptors, suggestive of energetically favorable access to the active state (Supplementary Table 1). Nevertheless, their mean channel conductance is similar to that of WT, despite a modest reduction (6.0 ± 0.3 pS vs. 8.0 ± 0.5 pS), indicating similar conductance properties upon channel opening (Extended Data Fig. 3a-c). In the absence of CGP, the GluN3A^{ELSL}-containing receptor displays nearly complete desensitization, albeit with slower onset ($\tau_{D,W}$ 102 ± 8 ms) and*

faster recovery from desensitization ($\tau_{recovery}$ 3.2 ± 0.1 s) compared to WT ($\tau_{D,W}$ 34 ± 4 ms, $\tau_{recovery}$ 5.5 ± 0.7 s) (Extended Data Fig. 3d-g and Supplementary Table 1), consistent with the mutations 'strengthening' the D1-D1 interface. Thus, the GluN3A^{ELSL}-containing receptor retains WT-like receptor function in terms of activation and desensitization in the absence of CGP. We therefore utilized GluN1$_{EM}$/GluN3A^{ELSL}$_{EM}$ receptors to determine an activated, open channel conformation".

We have added a new Extended Data Fig. 3 accordingly, as shown below.

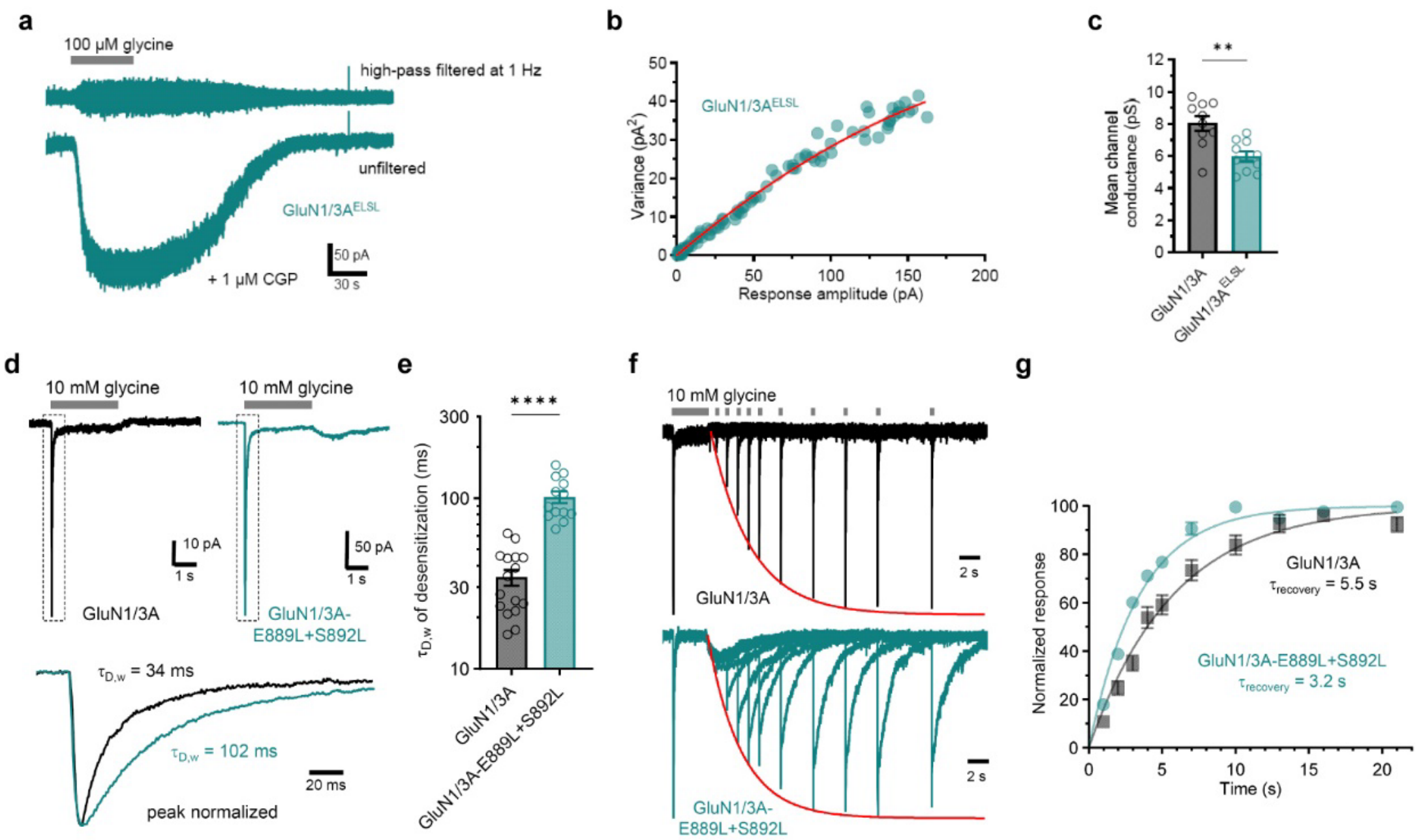

Extended Data Fig. 3: GluN1/GluN3A^{ELSL} receptors retain activation and desensitization similar to WT receptors. a, Representative whole-cell patch-clamp recording of a response from GluN1/3A-E889L+S892L (GluN1/GluN3A^{ELSL}) receptors to bath-applied 100 μM glycine in the continuous presence of 1 μM CGP-78608 with the same recording high-pass filtered at 1 Hz shown on top to illustrate noise arising from channel gating. **b**, Representative current-variance plot corresponding to the recording shown in panel a. **c**, The bar graph summarizes mean channel conductance measured from cells expressing WT (8.0 ± 0.5 pS, n = 10) or ELSL mutant (6.0 ± 0.3 pS, n = 10) GluN1/3A receptors (** $p < 0.01$; unpaired t-test). **d**, Representative recordings showing WT and ELSL mutant GluN1/GluN3A receptor desensitization in the absence of CGP-78608, overlay of responses highlighted by dashed boxes. Responses were activated by 3 s exposures to 10 mM glycine. **e**, The bar graph summarizes weighted desensitization time constants ($\tau_{D,w}$) (GluN1/3A, n = 16; GluN1/3A^{ELSL}, n = 13) (**** $p < 0.001$; unpaired t-test). **f**, Representative recordings illustrating recovery from desensitization following a 3 s exposure to 10 mM glycine. Each recording is made from 10 sweeps with a long 3 s exposure at the beginning followed by a 10 ms test pulse at different intervals following the long exposure. The red line represents the fit to response amplitudes as a function of time from the end of the long exposure to the test pulse. **g**, The graph summarizes the time course of recovery from desensitization (GluN1/3A, n = 7; GluN1/3A^{ELSL}, n = 7). Data are shown as mean ± SEM. See Supplementary Table 1 for parameters describing functional properties.

We sincerely appreciate the comments related to MD studies and emphasize that we have substantial, in depth experience with simulations on NMDA receptors (see PMID: 29670280). As we found in the previous work, meaningful MD simulations require long time scales and very substantial computational requirements, and are major, independent research projects in-and-of themselves. Such computational investigations, as well as additional experimental work, will form the basis for future studies.

2. The identification of class 3 as an "open" state is based on an entirely unresolved TMD density and inferred M3 displacement, but no full atomic model is presented. The pore dimensions and ion conduction compatibility cannot be quantitatively validated (e.g., via pore radius analysis). Without electrophysiological correlation to specific structural states (e.g., via state-selective trapping or conductance measurements), assignment of the open state remains inferential. The absence of resolved selectivity filter conformational changes in the open state limits mechanistic certainty. The authors should provide additional structural, or MD simulation-based evidence that the observed state indeed adopts an "open" conformation. Finally, have the authors considered the possibility that this state is not, in fact, C2 symmetric, and the TMD is poorly resolved because C2 symmetry was applied?

We have clarified the limitation of the unresolved TMD structure in the Discussion section (lines 446–448): "*Lastly, the diffuse TMD density observed in the active state (class 2 and 3) GluN1/GluN3A cryo-EM maps limited reliable model building, precluding structural analysis*".

Regarding electrophysiological correlation to the open states, as mentioned above, we have added unitary conductance data from WT and mutant receptors, showing that the ELSL receptor visits an open state that is similar to the WT receptor (Extended Data Fig. 3a-c in the revised manuscript).

For symmetry application, the cryo-EM map was ultimately refined without imposed symmetry. We initially reconstructed the map in C1, and C2 symmetry was applied to improve TMD features, then the final map used for analysis was obtained after C2 symmetry relaxation. Importantly, the overall features of the TMD were consistent across C1, C2, and symmetry-relaxed reconstructions.

3. The SiMPull data suggest that the predominant native assembly in neonatal mouse brain is diheteromeric GluN1/GluN3A. However:
Approximately one-third of complexes show single-step photobleaching. The study does not rule out the presence of triheteromeric GluN1/GluN2/GluN3A assemblies in other developmental stages or brain regions. Given the physiological importance of triheteromeric assemblies reported in prior literature, the authors should more carefully delimit their conclusions to the specific developmental stage and preparation analyzed.

We agree that our SiMPull analysis does not exclude the presence of GluN3A-containing receptors with alternative stoichiometries, including potential triheteromeric GluN1/GluN2/GluN3A assemblies. We have revised the sentences (lines 108-110) to clarify this point, which reads "*The remaining ~33% of the fluorophores exhibited one-step bleaching, likely due to incomplete antibody binding, pre-bleaching or degradation of the fluorophore prior to detection, and the potential presence of triheteromeric GluN1/GluN2/GluN3A assemblies*".

4. Antibody 5E3: The novel antibody 5E3, which specifically targets GluN3A is poorly described.

How was it isolated/engineered and what is the epitope? Is it possible that the antibody skews the stoichiometry towards specific heteromers?

The 5E3 antibody was generated by immunizing mice with proteoliposomes containing $GluN1_{EM}$/$GluN3A_{EM}$ receptors (without GFP) used in this study. The antibody was isolated from hybridoma clones and further characterized for subunit specificity, with cross-reactivity assessed as described in the cited reference (ref. 43). Because the receptor lacked the cytosolic domains, 5E3 is expected to recognize an extracellular epitope of GluN3A, most likely within the amino-terminal domain (ATD), which shows low sequence identity (~22%) to the corresponding regions of other NMDAR subunits. While this antibody was extensively characterized using recombinantly expressed receptors, we cannot exclude the possibility that antibody binding may influence the observed stoichiometry in mouse brain samples, and we have noted this as a potential limitation in the revised manuscript (lines 440-443), which reads "*In addition, the epitopes for GluN3A antibody binding are not yet fully defined, although the antibodies likely bind to the extracellular region of the receptor. Differences in cellular context and the effects of antibody binding may influence receptor assembly and the observed stoichiometry, both of which are limitations of this study*".

5. Naturally, a large emphasis of the paper is focused on symmetry of the respective reconstructions (2fold and 4fold), while very little information is provided about the exact details regarding processing with symmetry applied. Have the authors attempted C1 reconstructions for all maps? How confident are they that symmetry is indeed present and not just assumed?

The 2-fold and 4-fold symmetric arrangements described in the manuscript refer to the structural organization of the LBD and TMD layers arising from their subunit arrangements, rather than symmetry imposed during cryo-EM reconstruction in CryoSPARC. All cryo-EM maps presented in this study were reconstructed in C1, and the atomic models were built mostly using these C1 reconstructions. C2 symmetry was applied only when it improved quality of the resulting density maps and, even then, the fitting of the model to the maps was always carried about by reference to both C1 and C2 symmetry applied maps. Thus, the structural features described in the manuscript are supported by reconstructions performed without imposed symmetry. Details of the processing workflow, symmetry application, and modeling are available in Extended Data Figs. 4, 6, and 8, as well as in Supplementary Table 2 and the Methods section.

6. The 'neural network'/cryoDRGN based analysis does not appear of very high quality. The movies provided show very low resolution reconstructions, and require lowering thresholds significantly, to see the respective densities. Ultimately the changes within these movies seem rather stochastic and densities appear and disappear, rather than showing clear conformational trajectories/deformations. Additional validation through other software packages (or established ones) would be critical to validate these findings.

We also performed other software packages such as 3D Variability Analysis and 3D Flex in CryoSPARC in addition to the cryoDRGN approach. However, these software did not yield stable or interpretable results, perhaps because of differences in underlying modeling methods. In our experience, cryoDRGN provided the most consistent and interpretable representation of the ATD mobility among these analyses. Nevertheless, the point of these analyses is to show that the conformational heterogeneity in the ATD layer is better described by continuous motion rather than

a few discrete states. In this context, the lower quality/resolution of the ATD layer compared to the LBD–TMD layer is reasonable, and lower map thresholds are required to visualize the ATD densities in the cryoDRGN movies. This observation is consistent with the limited resolution of the ATD densities reported in this and previous studies. We have noted this limitation of the ATD reconstruction in the revised manuscript lines 444-446, which reads "*Additionally, the ATD in the GluN1/GluN3A cryo-EM maps remained unresolved due to conformational mobility, preventing model building and structural analysis of this region*".

Minor points:
1. What was the rationale for not running a local refinement on the 'pre-active' state structure? All other states appears to have separate refinements for LBD and TMD.

We performed local refinement of each LBD and TMD layer for the pre-active state as well. However, the resulting maps showed similar quality to the LBD-TMD reconstruction, with differences in resolution of only <0.1 Å. This likely reflects the relatively stable conformation of the pre-active state, such that separate layer refinements do not substantially improve map features. In contrast, for the other states, local refinements led to noticeable improvements in map quality and model interpretation, therefore those results are reported in the manuscript.

Reviewer #4 (Remarks to the Author):

This paper reports a substantial body of new information on NR3 subunit NMDA receptors, which paradoxically are activated by glycine and not glutamate. These receptors reach peak expression during early development, when they regulate synapse development, but their molecular structure and function is the least well understood amongst the NMDA receptor family. I anticipate that the study will be of interest to both the large community working on glutamate receptors and also to membrane protein structural biologists working on ion channels.

Comments

The key findings are

(1) The first definition, using single molecule pulldown and photobleaching, of a stoichiometry of 2NR1/2NR3A for native receptors purified from CNS tissue. The data clearly shows that this is the major species, with only a small population of GluN3A receptors with a potentially different stoichiometry. This 2:2 stoichiometry was validated in structural studies on recombinant receptor assemblies.

(2) Establishment of a structurally based gating scheme from the resting, to pre-active, to active, to desensitized states, achieved via cryo-EM analysis of multiple structures under conditions designed to trap these states using a combination of agonists and antagonists, mutants, and allosteric modulators. Notably this substantially extends our understanding of the gating cycle, which was not possible based on prior work, with the caveat that the ion channel structure in the

active state is not well resolved.

(3) The first resolution of the ion channel structure (in the resting and pre-active states) including the pore helix and selectivity filter. This is a major step forwards and explains the low Ca permeability, and attenuated block by Mg that characterize NR3 activity, and the distinct V-gate that seals the pore in the closed state. Likewise, resolution of the LBD-TMD linker density reveals unique structures that underlie gating in NR1/NR3A assemblies, and how these are distinct from that in NR1/NR2B. All of these features, i.e. both pore and linker structures, were unresolved in prior work by Michalski & Furukawa.

(4) Identification of novel binding sites for two allosteric modulators that stabilize the open state, opening the field for design of novel ligands.

(5) Resolution of a long-standing paradox for the mechanism by which the NR1 glycine site competitive antagonist "CGP" potentiates the activation of NR1/NR3A, with steric hindrance by the CGP bound NR1 LBD open cleft conformation preventing rotation of the LBD assembly into the 4-fold symmetric desensitized state previously found for kainate receptors.

The paper is well written and the experiments and analysis of exceptionally high quality. Of note, an impressive number of functional experiments are performed using receptors with structure informed mutations (e.g. Fig 7 and Extended data Figs 8 and 9). Overall, while comparison with the 2024 Michalski & Furukawa paper is inevitable, the much more comprehensive body of data reported here represents a landmark study that firmly puts our understanding of NR3 receptors on a solid basis. Discussion of prior work is appropriate and fair. Of interest, using a recently developed neural-network reconstruction process, it is shown that the ATD assembly of NR1/NR3A receptors is highly dynamic, sampling a continuum of conformations, preventing accurate structural analysis of this region, as confirmed by Q-score analysis of prior NR1/NR3A receptor structures, and the marginal quality of previously reported maps for this region.

In my opinion the paper tics all the boxes for NSMB.

Minor comments

Title: Because GluN1/GluN2 receptors are also glycine-dependent, perhaps change "glycine-dependent NMDA receptors", to glycine-activated NMDA receptors?

We have revised the title as suggested.

Line 158: Rephrase 'primarily distinguished by their TMD conformations'. This is a bit tricky given the incompletely resolved TMD density for the active state, and absence of a structural model to define an active conformation. Extended data Fig 6 compares raw electron density, but does not show structures. The more detailed discussion of the active state on pages 12-13 more accurately describes the data and its limitations.

We have revised the sentence (lines 168-170) to read "*Under the CGP/Gly/GNE/UCM condition, we identified two major conformational classes that we deem pre-active and active, distinguished by conformational changes associated with the LBD layer, as described in a later section*".

We also agree with the reviewer that more detailed discussion of the active state and its limitations is required. Accordingly, we have substantially revised this paragraph (lines 227-233) to tone down the overall interpretation of class 3 and to limit comparisons with other glutamate receptors. We have also renamed this class as a second active conformation ("active 2"), based on its similarity in LBD conformation to the active state (class 2). The paragraph now reads: "*Further single particle cryo-EM image processing of receptor complexes in the presence of CGP/Gly/GNE/UCM uncovered another 3D class, deemed class 3, in which portions of the TMD are partially resolved. Notably, class 2 and class 3 adopt indistinguishable LBD conformations. Comparison with the well-defined densities in the pre-active state (class 1) suggests features of the TMD density in class 3. However, the low-resolution density of the class 3 TMD is not sufficient for building an atomic model, thereby limiting further structural interpretation (Extended Data Fig. 7d,e). We therefore suggest class 3 as a second active conformation (active 2)*".

We have described this limitation in the revised manuscript lines 446-448, which reads "*Lastly, the diffuse TMD density observed in the active state (class 2 and 3) GluN1/GluN3A cryo-EM maps limited reliable model building, precluding structural analysis and comparison of the TMD in the active state across different functional states*".

Line 238: clarify if the 21.4° degree rotation refers to the lower lobe of the LBD, or does the whole GluN3A ELSL mutant LBD (including the upper lobe) rotate? If the latter case is true, is it possible to distinguish whether the conformational differences are in part due to the ELSL mutant versus the wild type antagonist bound structure, or to the binding of glycine to the GluN3A subunit? It would be useful to have a structure of the antagonist bound the ELSL mutant for comparison.

The rotation refers to the whole GluN3A ELSL mutant LBD bound to glycine. While it is possible that some local conformational differences arise from the ELSL mutation, the mutant receptor also exhibits nearly complete desensitization upon glycine binding in the absence of CGP, consistent with that of the wild-type receptor, as well as reduced but not significant changed unitary conductance (Extended Data Fig. 3 in the revised manuscript). Although a structure of the antagonist-bound ELSL mutant receptor could be informative, this may be challenging as our previous attempts to incorporate the antagonist (DCKA) into the wild-type GluN3A receptor were unsuccessful, even at a high concentration. We have revised the sentences (line 240) in the manuscript to read "*Upon comparing the pre-active state of the GluN1-GluN3A^{ELSL} complex with the wild-type-like antagonist-bound state we find that the whole GluN3A^{ELSL} LBD has undergone a 21.4° rotation toward the GluN1 LBD (Fig. 3c)*".

Line 274: the 4-fold symmetric structure for the kainate receptor desensitized state was first reported by Schauder et al 2013, PNAS PMID: 23530186 and should be cited here.

We have added this citation.

Line 289: the authors speculate, reasonably, that the ELSL mutant stabilizes the D1 dimer assembly; experimental support for this hypothesis is reported in Table 1 which reports a 4-fold

slower time constant for desensitization the ELSL mutant compared to that for wild type GluN1/GluN3A. This should be reported in the main text.

We have added this point in the revised manuscript lines 156-160, which reads "*In the absence of CGP, the GluN3A^{ELSL}-containing receptor displayed nearly complete desensitization, albeit with slower onset ($\tau_{D,W}$ 102 ± 8 ms) and faster recovery from desensitization ($\tau_{recovery}$ 3.2 ± 0.1 s) compared to WT ($\tau_{D,W}$ 34 ± 4 ms, $\tau_{recovery}$ 5.5 ± 0.7 s) (Extended Data Fig. 3 and Supplementary Table 1)*".

Line 389: 'remains unbound'. This needs justification or revision. 5,7-DCKA was present at 5 mM and would be anticipated to bind, but not with full occupancy, based on its published affinity of 650 μM for GluN3A; perhaps the absence of density might be due to partial occupancy? Was weak density observed with a different contour level?

Although 5,7-DCKA was present at a high concentration during cryo-EM grid preparation, we did not observe density corresponding to DCKA in the LBD-focused refinement or at different contour levels of the cryo-EM map. This discrepancy in DCKA affinity may be associated with differences in experimental context, particularly between isolated GluN3A LBD and the full receptor complex. The phrase 'remains unbound' was intended to mean the absence of agonist/antagonist binding. We have revised this sentence to read "*…the GluN3A clamshell remains unoccupied and the D1-D1 interface is partially disrupted*".

Line 416: for clarity it would be useful to state: we cannot exclude the occurrence of minor populations of GluN3A receptors with different stoichiometries, but note that di-heteromeric GluN1/GluN3A receptors predominate.

We have included this point in the relevant Results section of the SiMPull analysis (lines 108-110), which reads "*The remaining ~33% of the fluorophores exhibited one-step bleaching, likely due to incomplete antibody binding, pre-bleaching or degradation of the fluorophore prior to detection, and the potential presence of triheteromeric GluN1/GluN2/GluN3A assemblies*".

Line 418 missing references (38-40) See also comment for line 274.

Thank you for this comment. We have added these citations.

Line 719 FSEC was performed

Corrected.

Line 987 sharpen should be sharpened, line 991 A low pass filter, and line 995 unsharp should be unsharpened

Corrected.

Figure 2a legend: state the duration of the glycine applications for the left and right hand panels; same for panel c. Same for Extended data Fig. 8 d and h.
We have added this information.

Supplementary Fig 1 panel C. The large difference in Q scores across the structures would be better

emphasized if the Y-axis was plotted using the same scale for all panels, i.e. 0.8 to -0.6, with a dotted line at zero in all panels. Likewise, the X-axis should be from 0 to 950 in all panels, to fairly compare the Q-scores in the LBD segment.

We have revised the figure as suggested.
